# The clinical prognostic value of long noncoding RNA HAND2-AS1 in cancer patients: A study based on meta-analysis and TCGA data (PRISMA)

**DOI:** 10.1097/MD.0000000000030789

**Published:** 2022-09-30

**Authors:** Zhaoyang Yan, Juntao Lu, Xinjian Xu, Yang You, Jinsheng Xu, Tongxin Xu

**Affiliations:** a Department of Thoracic Surgery, The Fourth Hospital of Hebei Medical University, Shijiazhuang, Hebei Province, China; b Laboratory of Pathology, Hebei Cancer Institute, The Fourth Hospital of Hebei Medical University, Shijiazhuang, Hebei Province, China; c Department of CT&MRI, The Fourth Hospital of Hebei Medical University, Shijiazhuang, Hebei Province, China; d Hebei Key Laboratory of Vascular Calcification in Kidney Disease, Hebei Clinical Research Center for Chronic Kidney Disease, The Fourth Hospital of Hebei Medical University, Shijiazhuang, Hebei Province, China.

**Keywords:** cancer, HAND2-AS1, long noncoding RNA, overall survival, prognosis

## Abstract

The heart and neural crest derivatives expressed 2 antisense RNA 1 (HAND2-AS1) is a novel long noncoding RNA aberrantly expressed in human malignancies. We aimed to analyze the available data to evaluate the clinical prognostic significance of HAND2-AS1 in tumors.

**Methods:**

In this meta-analysis, electronic databases, including PubMed Cochrane Library, EMBASE, Medline, Web of Science, CNKI, and Wanfang, were searched from their inception up to December 1, 2021. The pooled hazard ratios (HRs) or odds ratios (ORs) with 95% confidence intervals (95% CIs) were calculated to assess the relationship of HAND2-AS1expression level with prognosis and clinicopathological features in cancer patients. The publication bias was identified by Begg’s test, and the sensitivity analysis was also performed.

**Results:**

A total of 10 articles with 615 patients were included in the present meta-analysis. The combined results revealed that low expression of HAND2-AS1 was associated with poor overall survival (OS) (HR = 0.48, 95% CI: 0.36–0.64, *P* < .001) in a variety of cancers. In addition, the decrease in HAND2-AS1 expression was also correlated with poor differentiation (OR = 4.36, 95% CI: 2.15–8.87, *P* < .001) and lymph node metastasis (OR = 0.26, 95% CI: 0.13–0.54, *P* < .001). The cancer genome atlas (TCGA) dataset further demonstrated that low expression of HAND2-AS1 was associated with poor OS and disease-free survival.

**Conclusions:**

Our results of this meta-analysis indicated that HAND2-AS1 may be a prognostic marker and even a therapeutic target for human cancer.

## 1. Introduction

Cancer ranks as a leading cause of death and an important barrier to increasing life expectancy in the world.^[1]^ According to GLOBOCAN, there were 19.3 million new cases and 10 million cancer deaths worldwide in 2020.^[2]^ Despite the significant progress made in treatment, including chemotherapy, radiotherapy, and surgical techniques, patients with advanced-stage cancer still have a poor prognosis.^[3]^ Therefore, it is very meaningful to early diagnose tumors, especially finding a novel molecular cancer biomarker that correlates with cancer progression and prognosis.^[4]^

Long noncoding RNA (lncRNA) is a kind of noncoding RNA that has little or no ability of coding protein and is longer than 200 base pairs.^[5]^ Over the past few years, lncRNA has been the research focus in biology. An amount of evidence shows that lncRNA could function as master regulators for gene expression and play a vital role in various physiopathologic and disease processes including cancer.^[6,7]^ Dysregulated lncRNA expression was found in many human tumors, suggesting that lncRNA has the potential to be a diagnostic and prognostic indicator.^[8,9]^

Heart and neural crest derivatives expressed 2 antisense RNA 1 (HAND2-AS1) is a newly identified lncRNA located in the chromosome 4q33-34 region and transcribed in a head-to-head orientation of HAND2.^[10]^ Overexpression of this lncRNA could significantly inhibit many phenotypes such as proliferation, migration, invasion, and energy metabolism in the process of tumor development.^[11]^ Several studies have indicated that HAND2-AS1 is downregulated in various tumor tissues, such as hepatocellular,^[12]^ bladder,^[13]^ lung,^[14]^ gastric,^[15]^ colorectal,^[16,17]^ osteosarcoma,^[18]^ breast,^[19]^ ovarian,^[20]^ cervical,^[21,22]^ and endometrial.^[23]^ Moreover, most existing data suggest that HAND2-AS1 is correlated with cancer patient survival and may be a prognostic biomarker. However, these studies are limited by their relatively small sample size. Therefore, we performed a meta-analysis to explore the clinical prognostic value of the lncRNA HAND2-AS1 in patients with cancer.

## 2. Methods

### 2.1. Literature search

Two authors independently searched the electronic databases including PubMed, Cochrane Library, EMBASE, Medline, Web of Science, CNKI, and Wanfang from their inception up to December 1, 2021, to identify potential studies. Both MeSH terms and free-text keywords were used for searching relevant articles. The terms were listed as follows: (“heart and neural crest derivatives expressed 2 antisense RNA 1” or “HAND2-AS1”) AND (“neoplasm” or “cancer” or “tumor” or “carcinoma” or “sarcoma” or “malignancy”). An additional manual search of the reference lists of all potentially eligible articles was performed.

### 2.2. Inclusion and exclusion criteria

The studies were included based on the following criteria: patients were diagnosed with cancer and divided into two groups based on the expression of HAND2-AS1; the relationship between HAND2-AS1 expression levels and clinicopathological features or survival were described; sufficient original data to calculate the pooled hazard risk (HR) and 95% confidence interval (CI).

The criteria for exclusion were as follows: studies without clinical outcomes or prognosis of cancer; duplicate publications; animal studies, letters, editorial, abstracts, case reports, or reviews.

### 2.3. Data extraction and quality assessment

Data extraction was performed independently by two authors, and different opinions were resolved by discussion with the third author. The following data from the included studies were extracted: author, year of publication, country, tumor type, TNM stage, sample size, patients’ number of high HAND2-AS1 expression group and low HAND2-AS1 expression group, type of survival analysis, follow-up time, hazard ratio (HR) and its confidence interval (95% CI), and the relationship between HAND2-AS1 expression and clinicopathological features of the patients.

If only the Kaplan–Meier survival curves were available, we extracted the information from the graph survival plot and calculated HRs with 95% CIs via Engauge Digitizer software (Version 4.1). If the HRs, 95% CIs, and *P* values from the multivariate analyses for overall survival (OS) were reported, there were directly collected. The quality of each eligible study was evaluated using Newcastle–Ottawa quality assessment scale (NOS) scores and studies with a NOS score ≥6 were considered high quality.^[24]^

### 2.4. Bioinformatics analysis of the cancer genome atlas (TCGA) data

The TCGA-based visualization website GEPIA (http://gepia.cancer-pku.cn/) was used for the analysis of the expression level of HAND2-AS1 in various tumor types. Further evaluations of the associations between HAND2-AS1 level with OS and disease-free survival (DFS) were also conducted. Differential expression analysis was carried out via one-way ANOVA. Kaplan–Meier (K–M) method and log-rank test were employed to perform survival outcome analysis. HRs and *P* value were presented as K–M curves.

### 2.5. Statistical analysis

All statistical analyses were performed via Review Manager (RevMan 5.3) and STATA software (Version 12.0). The pooled HRs with the corresponding 95%CIs were calculated to analyze the association of HAND2-AS1 expression with survival outcomes. HR <1 indicated that lncRNA overexpression predicted longer survival. The relationship between HAND2-AS1 expression level and clinicopathological parameters was estimated using the aggregated odds ratios (ORs) with 95% CIs. The heterogeneity among different studies was determined using the chi-square-based Cochran Q test and *I*^2^ statistic. The fixed-effects model was applied for data analysis when *I*^2 ^< 50%. Otherwise, the random-effects model was used for data analysis.

Sensitivity analysis was performed by sequential omission of individual studies to verify the stability of outcomes in this meta-analysis. The potential publication bias was estimated via Begg’s funnel plot and Egger’s test. *P* < .05 was considered statistically significant.

### 2.6. Ethics and dissemination

The ethical approval and patient consent were waived in this secondary research evidence. The results of this study will be disseminated through peer-reviewed academic journals and conferences.

## 3. Results

### 3.1. Characteristics and basic information in eligible literature

**Figure 1.**
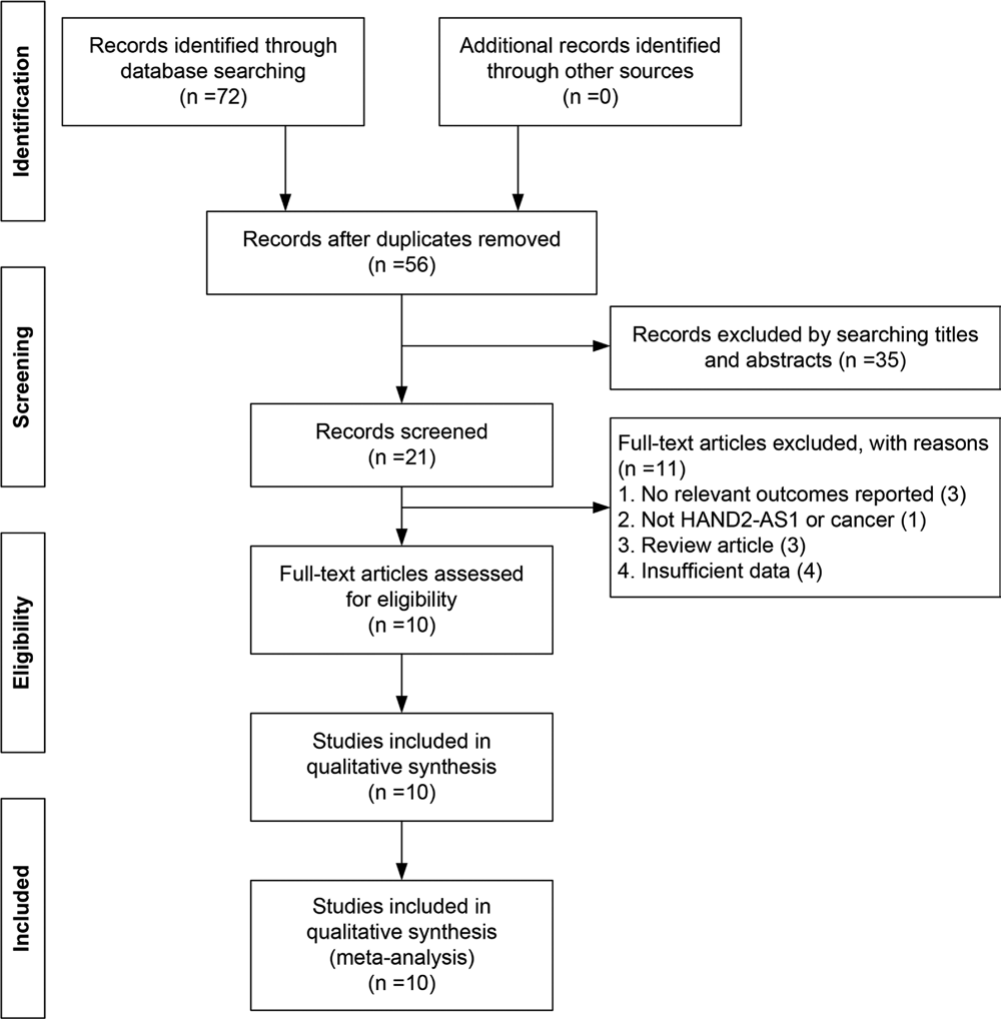
Flow diagram of the study selection procedure in this meta-analysis.

According to the pre-established research strategy, we preliminarily retrieved 72 relevant studies. After carefully reading the title, abstract, and full text, a total of 10 articles with 615 patients were included in the current meta-analysis.^[12–19,21,22]^ The flow diagram of study search and selection was summarized in Figure 1. The expression of HAND2-AS1 was detected by quantitative real-time PCR in all studies. As shown in Table 1, all studies were performed in China in a publication period between 2018 and 2021. Eight different tumor types were included: colorectal cancer (n = 2), cervical cancer (n = 2), hepatocellular cancer (n = 1), bladder cancer (n = 1), gastric cancer (n = 1), lung cancer (n = 1), breast cancer (n = 1), and osteosarcoma (n = 1). The NOS scores of the included studies ranged from 6 to 8, indicating that these studies were of high quality (Table 1).

**Table 1:** The main characteristics of the eligible literature included in the meta-analysis.

Study	Yr	Country	Tumor type	TNM stage	Sample size	HAND2-AS1 expression		Survival outcome	Follow–up (mo)	NOS score
						High	Low			
Bi et al^[12]^	2020	China	Hepatocellular cancer	NA	50	25	25	OS	72	7
Chen et al^[18]^	2019	China	Osteosarcoma	I-IV	48	24	24	NA	NA	6
Gao et al^[14]^	2020	China	Lung cancer	I-IV	94	47	47	OS	60	8
Gao et al^[21]^	2021	China	Cervical cancer	I-II	58	29	29	OS	60	8
Gong et al^[22]^	2020	China	Cervical cancer	I-III	57	29	28	OS	35	6
Jiang et al^[16]^	2020	China	Colorectal cancer	NA	50	25	25	OS	60	7
Shan et al^[13]^	2021	China	Bladder cancer	I-IV	32	9	23	OS	60	7
Wang et al^[19]^	2020	China	Breast cancer	NA	62	26	36	OS	60	6
Xu et al^[15]^	2020	China	Gastric cancer	I-IV	90	19	71	NA	NA	6
Zhou et al^[17]^	2018	China	Colorectal cancer	I-IV	74	37	37	OS	150	8

NA = not available, NOS = Newcastle–Ottawa quality assessment scale, OS = overall survival, TNM = tumor node metastasis.

### 3.2. HAND2-AS1 expression significantly correlated with OS

**Figure 2.**
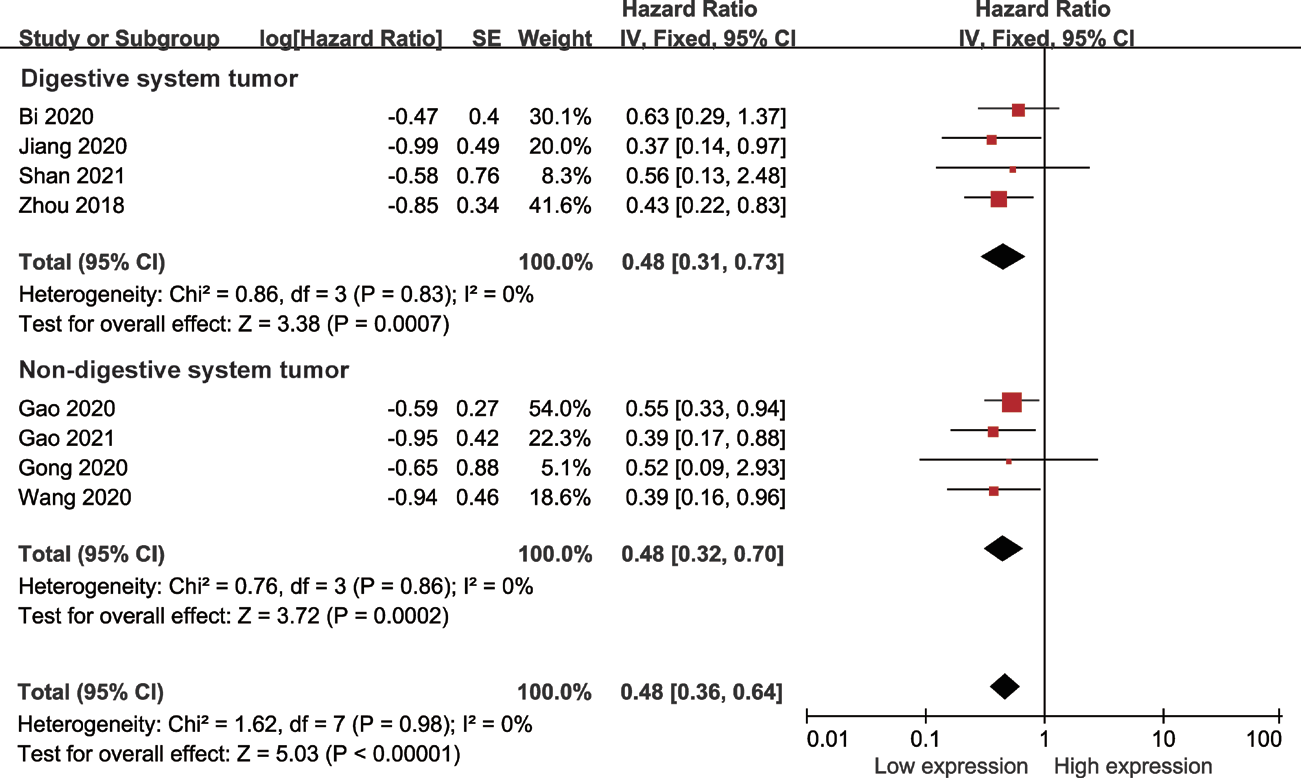
Forest plots of the included studies evaluating the association between HAND2-AS1 expression with overall survival (OS).

Eight studies including 477 patients assessed the HR and 95% CI of OS. As shown in Figure 2, the fixed-effect model was applied to calculate the pooled HR and its 95% CI due to no significant heterogeneity among the studies (*I*^2^ = 0%, *P* = .98). The pooled result showed that low expression of HAND2-AS1 predicted unfavorable OS in cancers (HR = 0.48, 95% CI: 0.36–0.64, *P* < .001). Subgroup analyses were also conducted based on tumor type (digestive system tumors and non-digestive system tumors). The results indicated that low expression of HAND2-AS1 was strongly correlated with the poor OS in both subgroups (digestive system tumors: HR = 0.48, 95% CI: 0.31–0.73, *P* < .001; non-digestive system tumors: HR = 0.48, 95% CI: 0.32–0.70, *P* < .001).

### 3.3. Association between HAND2-AS1 and clinicopathologic characteristics

**Figure 3.**
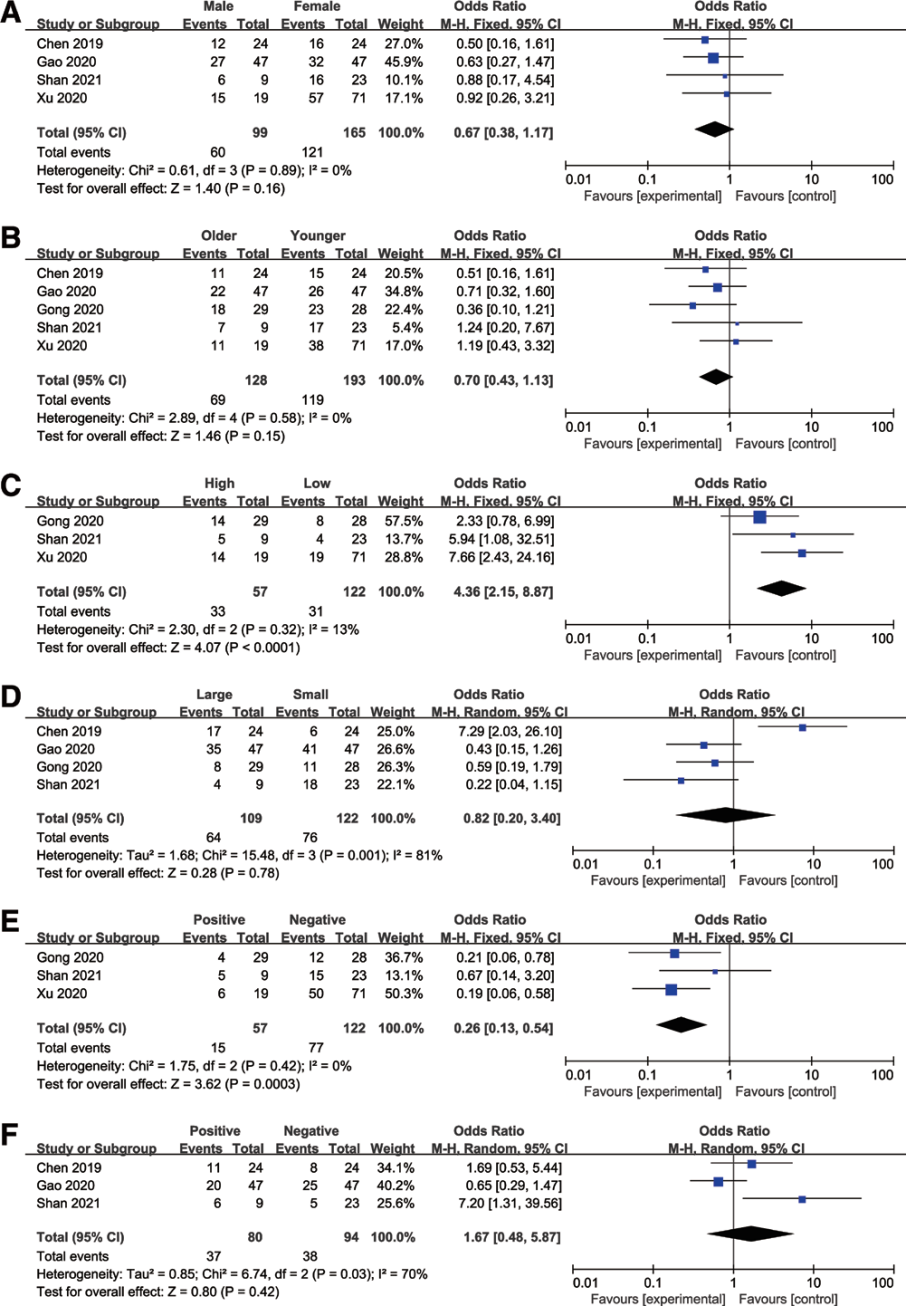
Forest plots of the included studies evaluating the correlation between HAND2-AS1 expression and clinicopathological characteristics: (A) gender; (B) age; (C) differentiation; (D) tumor size; (E) lymph node metastasis; (F) distant metastasis.

We further investigated the relationship of HAND2-AS1 expression with clinicopathologic parameters of cancer patients. As shown in Figure 3, low expression of HAND2-AS1 was related to poor tumor differentiation (OR = 4.36, 95% CI: 2.15–8.87, *P* < .001) and lymph node metastasis (OR = 0.26, 95% CI: 0.13–0.54, *P* < .001). Nevertheless, for the other clinical parameters, including gender, age, tumor size, and distant metastasis, no significant correlation was found between the HAND2-AS1 expression and the parameters. The details are presented in Table 2.

**Table 2:** The correlation between clinicopathological characteristics and HAND2-AS1 expression.

Clinicopathological parameters	Studies	Patients	OR (95% CI)	*P* value	Heterogeneity		
					*I*^2^ (%)	*P* value	Model
Gender (male vs female)	4	264	0.67 (0.38, 1.17)	.16	0	.89	Fixed
Age (older vs younger)	5	321	0.70 (0.43, 1.13)	.15	0	.58	Fixed
Differentiation (well and moderately vs poor)	3	179	4.36 (2.15, 8.87)	<.001	13	.32	Fixed
Tumor size (large vs small)	4	231	0.82 (0.20, 3.40)	.78	81	.001	Random
LNM (positive vs negative)	3	179	0.26 (0.13, 0.54)	<.001	0	.42	Fixed
DM (positive vs negative)	3	174	1.67 (0.48, 5.87)	.42	70	.03	Random

CI = confidence interval, DM = distant metastasis, LNM = lymph node metastasis, OR = odds ratio.

### 3.4. Sensitivity analysis and publication bias

**Figure 4.**
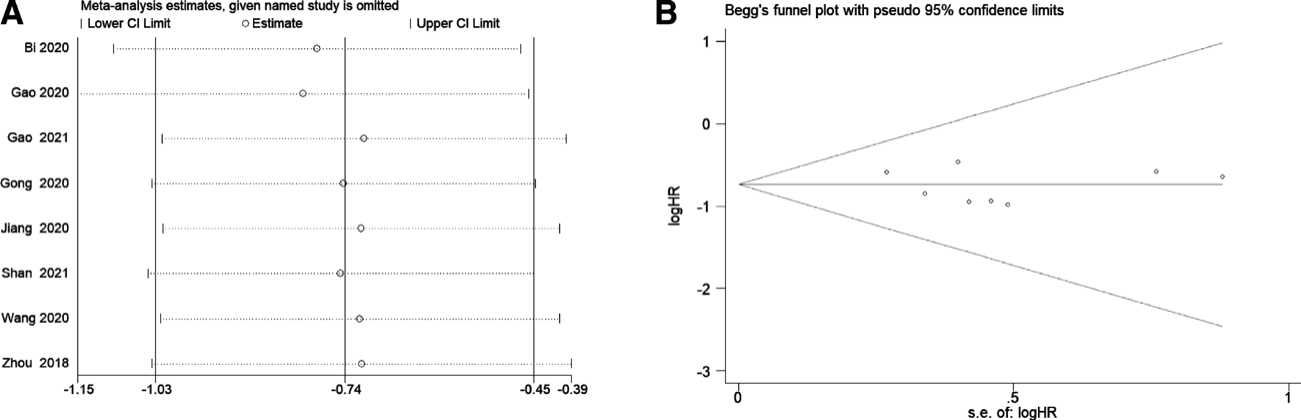
Sensitivity analysis and publication bias for OS in this meta-analysis. (A) sensitivity analysis; (B) Begg’s funnel plots.

Sensitivity analysis was performed by deletion of a single excluded study in each step. The results revealed that the pooled HR for OS was not altered significantly after deleting any eligible study, suggesting that our combined results are stable and reasonable (Fig. 4A). In addition, Begg’s funnel plot and Egger’s test were performed to evaluate the publication bias of the meta-analysis. Begg’s funnel plot was symmetrical, and Egger’s test showed *P* = .369, indicating that no significant publication bias for OS was measured (Fig. 4B).

### 3.5. Validation of the results in the TCGA dataset

**Figure 5.**
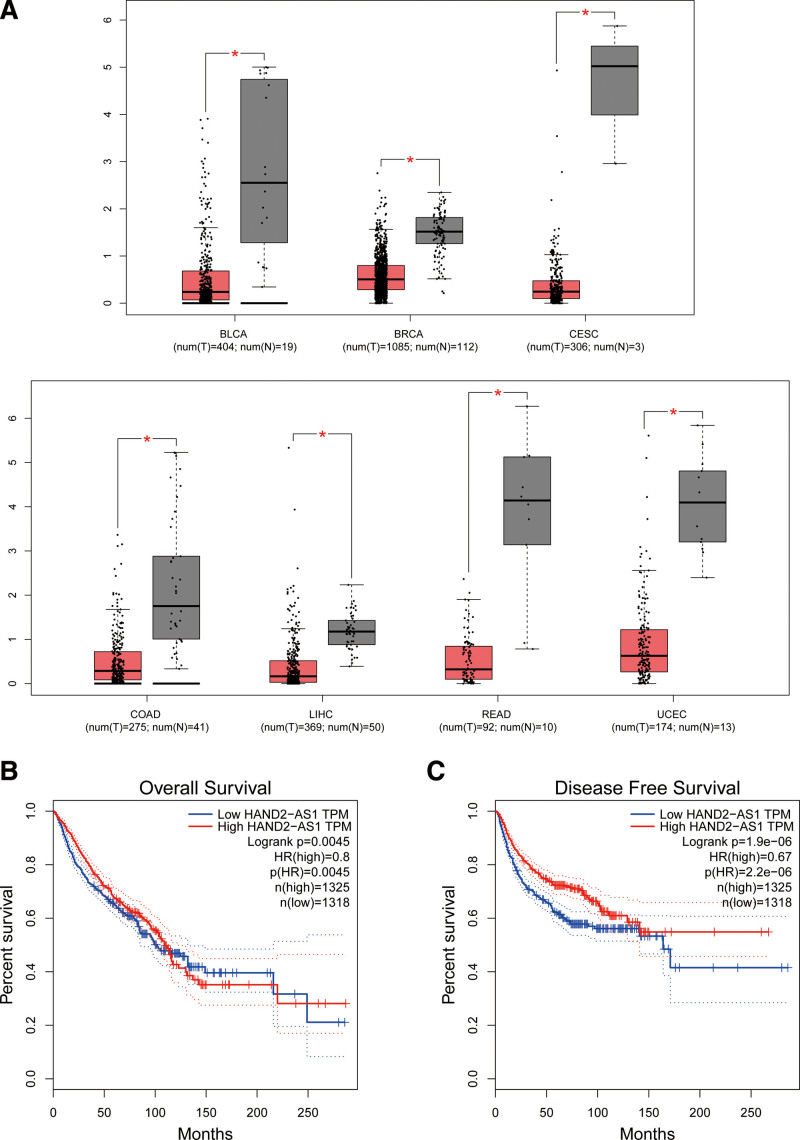
Validation of HAND2-AS1 expression in various cancers in TCGA cohort. (A) the expression of HAND2-AS1 in cancers and normal tissues; (B) overall survival plot of HAND2-AS1 in TCGA cohort; (C) disease-free survival plot of NNT-AS1 in TCGA cohort. TCGA (The Cancer Genome Atlas); BLCA (bladder urothelial carcinoma); BRCA (breast invasive carcinoma); CESC (cervical squamous cell carcinoma and endocervical adenocarcinoma); COAD (colon adenocarcinoma); LIHC (liver hepatocellular carcinoma); READ (rectum adenocarcinoma); UCEC (uterine corpus endometrial carcinoma).

Furthermore, we validated the expression of HAND2-AS1in the different types of cancers in The Cancer Genome Atlas (TCGA) database by bioinformatics analysis. As demonstrated in Figure 5A, HAND2-AS1 was significantly downregulated in bladder urothelial carcinoma, breast invasive carcinoma, cervical squamous cell carcinoma and endocervical adenocarcinoma, colon adenocarcinoma, liver hepatocellular carcinoma, rectum adenocarcinoma, and uterine corpus endometrial carcinoma when compared with control. Moreover, we merged the expression data and OS/DFS data of the above carcinomas from the TCGA dataset, which comprised 2643 patients categorized in high or low expression groups. The results showed that downregulated HAND2-AS1 expression predicted worse OS (HR = 0.80, *P* < .001), as well as DFS (HR = 0.67, *P* < .001), and these remained consistent with the consequences in the present meta-analysis (Fig. 5B and C).

## 4. Discussion

Increasing evidence has suggested that lncRNAs could play critical roles in almost all biological processes, including cell differentiation, proliferation, and apoptosis.^[25]^ Multiple studies have also revealed that lncRNAs could be potential cancer diagnostic biomarkers and therapeutic targets, such as HOTAIR,^[26]^ MALAT1,^[27]^ and TUG1.^[28]^ Previous studies have reported that HAND2-AS1 is a tumor-suppressor gene, and its low expression was correlated with aggressive clinicopathological features and unfavorable survival outcomes. However, due to the limited sample size and discrete outcomes, a meta-analysis of the published literature and bioinformatics analysis were performed to determine the clinical role of HAND2-AS1.

In this meta-analysis, ten studies with eight cancer types containing 615 patients were enrolled. The pooled results suggested that downregulated HAND2-AS1 closely correlated with shorter OS with no obvious heterogeneity existing in the analysis. The subgroup stratified analysis further demonstrated that the prognostic value of HAND2-AS1 for OS was not influenced by tumor type, suggesting that our combined results were reliable. Furthermore, no publication bias was detected between HAND2-AS1 expression and OS, highlighting the robustness of our results. The following bioinformatics analysis, on the basis of the TCGA cohort, also showed that HAND2-AS1 was obviously correlated with OS and DFS. In addition, the subsequent pooled results demonstrated that decreased HAND2-AS1 level was related to lymphatic metastasis and poor differentiation. These results indicated that HAND2-AS1 is a promising predictor for cancer.

Numerous researchers have tried to further explore the underlying mechanisms of HAND2-AS1 in carcinogenesis. A previous study found that the CpG122 and CpG74 hypermethylation in the promoter region of HAND2-AS1 led to its low expression.^[23]^ In bladder cancer, the HAND2-AS1/miR-146/RARB complex could promote Caspase 3-mediated apoptosis by suppressing COX-2 expression.^[13]^ Gong et al^[22]^ reported HAND2-AS1 could attenuate cell proliferation, migration, invasion, and tumorigenesis of cervical cancer by downregulating C16orf74 expression through recruiting E2F4. Yan et al^[29]^ demonstrated that HAND2-AS1 could enhance the inactivation of the JAK-STAT pathway to suppress liver cancer progression. In colorectal cancer, HAND2-AS1 could suppress cell progression and 5-FU resistance by upregulating PDCD4 via sponging miR-20a.^[16]^ Dong et al^[30]^ uncovered that HAND2-AS1 could suppress breast cancer cell growth by regulating the miR-3118/PHLPP2 signaling axis. In lung cancer, HAND2-AS1 was downregulated in both tumor tissue and plasma. Overexpression of HAND2-AS1 led to the significantly inhibited expression of TGF-β1 and reduced level of p-Smad2/3 in cancer cells.^[31]^ Chen et al^[18]^ showed that knockdown HAND2-AS1 could promote osteosarcoma cell proliferation and glucose uptake by upregulating GLUT1, which is a key protein of glucose metabolism. In gastric cancer, HAND2-AS1 could inhibit the glycolytic process induced by hypoxia through miR-184/HIF3A signaling.^[15]^ Taken together, the above evidence strongly supports that HAND2-AS1 may play a tumor suppressor role in human cancers.

Several shortcomings exist in this meta-analysis. First, all studies were performed in China, which may lead to geographical bias. Second, the number of studies focusing on a specific cancer type was relatively limited, so we could not aggregate results based on a single type of tumor. Third, the cutoff value for low or high levels of HAND2-AS1 varied in different studies, making it difficult to reach a consensus value. Forth, some HR values were not given directly in the literature. Thus, we calculated HR values manually from K–M curves, which may not be fully accurate. Therefore, large well-designed studies in multiple populations of different ethnicities and further functional studies are warranted to achieve a more definite conclusion.

## 5. Conclusions

In summary, low expression of HAND2-AS1 is significantly associated with unfavorable survival outcomes and worse clinicopathological characteristics in kinds of human tumors, suggesting that HAND2-AS1 may be a prognostic marker and even a therapeutic target. However, more prospective research and in-depth data analysis are needed to further confirm the value of HAND2-AS1 in cancer.

## Acknowledgments

The authors thank all authors of the included studies.

## Author contributions

**Conceptualization:** Zhaoyang Yan.

**Data curation:** Zhaoyang Yan, Juntao Lu, Xinjian Xu, Yang You, Tongxin Xu.

**Formal analysis:** Jinsheng Xu.

**Funding acquisition:** Juntao Lu, Tongxin Xu.

**Investigation:** Juntao Lu, Xinjian Xu.

**Methodology:** Xinjian Xu.

**Project administration:** Jinsheng Xu.

**Resources:** Jinsheng Xu.

**Software:** Zhaoyang Yan, Yang You.

**Supervision:** Jinsheng Xu, Tongxin Xu.

**Validation:** Yang You, Jinsheng Xu, Tongxin Xu.

**Visualization:** Yang You.

**Writing – original draft:** Zhaoyang Yan.

**Writing – review & editing:** Tongxin Xu.
